# Role of ALADIN in Human Adrenocortical Cells for Oxidative Stress Response and Steroidogenesis

**DOI:** 10.1371/journal.pone.0124582

**Published:** 2015-04-13

**Authors:** Ramona Jühlen, Jan Idkowiak, Angela E. Taylor, Barbara Kind, Wiebke Arlt, Angela Huebner, Katrin Koehler

**Affiliations:** 1 Klinik und Poliklinik für Kinder- und Jugendmedizin, Medizinische Fakultät Carl Gustav Carus, Technische Universität Dresden, Germany; 2 Centre for Endocrinology, Diabetes and Metabolism, School of Clinical & Experimental Medicine, University of Birmingham, United Kingdom; North Carolina State University, UNITED STATES

## Abstract

Triple A syndrome is caused by mutations in *AAAS *encoding the protein ALADIN. We investigated the role of ALADIN in the human adrenocortical cell line NCI-H295R1 by either over-expression or down-regulation of ALADIN. Our findings indicate that *AAAS* knock-down induces a down-regulation of genes coding for type II microsomal cytochrome P450 hydroxylases *CYP17A1 *and *CYP21A2* and their electron donor enzyme cytochrome P450 oxidoreductase, thereby decreasing biosynthesis of precursor metabolites required for glucocorticoid and androgen production. Furthermore we demonstrate that ALADIN deficiency leads to increased susceptibility to oxidative stress and alteration in redox homeostasis after paraquat treatment. Finally, we show significantly impaired nuclear import of DNA ligase 1, aprataxin and ferritin heavy chain 1 in ALADIN knock-down cells. We conclude that down-regulating ALADIN results in decreased oxidative stress response leading to alteration in steroidogenesis, highlighting our knock-down cell model as an important *in-vitro* tool for studying the adrenal phenotype in triple A syndrome.

## Introduction

Triple A syndrome (MIM*231550) is an autosomal-recessive disease manifesting with the triad of ACTH-resistant **a**drenal insufficiency, **a**chalasia of the cardia and **a**lacrima (**Triple A**) in combination with progressive neurological impairment [1]. The disease is caused by mutations in the *AAAS* (achalasia—adrenocortical insufficiency—alacrima syndrome) gene, which encodes the protein ALADIN (**al**acrima-**a**chalasia-a**d**renal **in**sufficiency neurologic disorder) [2,3]. *AAAS* is ubiquitously expressed, but shows an enhanced expression in the adrenal gland, gastrointestinal tract and pituitary gland [3]. In 2002, ALADIN was identified as a component of the nuclear pore complex (NPC) [4]. Human NPC is a large protein complex composed of approximately 30 different proteins, known as nucleoporins, which mediate the transport of macromolecules between the cytoplasm and the nucleoplasm [4]. Most of the known *AAAS* mutations result in mis-localisation of the altered ALADIN protein, mainly to the cytoplasm [5–7]. ALADIN is anchored within the NPC by the transmembrane nucleoporin NDC1 [8,9]. It belongs to the group of barely exchangeable nucleoporins and therefore seems to be a scaffold nucleoporin [10]. It is suspected that a dysfunction of ALADIN may play a role in cellular accumulation of reactive oxygen species (ROS). There is increasing evidence that ALADIN-deficient cells are more susceptible to oxidative stress [11–14]. During our ongoing investigations Prasad et al. recently published results on the involvement of ALADIN in oxidative stress response and steroidogenesis [12]. With our study we do not just reproduce results obtained by Prasad et al. but independently verify some of their aspects using an alternative cell model and present new results on the role of ALADIN for oxidative stress, steroidogenesis and nuclear import. The results discussed in this article therefore add to the understanding of the adrenal phenotype in triple A syndrome.

In addition, studying differential gene expression in ALADIN-deficient or-mutated cells under oxidative stress, we have previously seen that cells of triple A patients show an altered induction or down-regulation of genes associated with oxidative stress and antioxidant defence [13]. Ferritin heavy chain protein (FTH1) was identified as an interaction partner of ALADIN [14]. In addition to its well-known iron storage role, FTH1 has been shown to protect the nucleus from oxidative damage. It was hypothesised that mutant ALADIN impairs the nuclear import of FTH1 in triple A syndrome. A deficiency of nuclear FTH1 results in an enhanced susceptibility of cells to oxidative stress and cellular damage [14]. Furthermore Hirano et al. showed in a patient fibroblast cell line a reduced nuclear import of DNA ligase 1 (LIG1) and aprataxin (APTX) which both have functions in DNA single strand break repair, also demonstrating an increased sensitivity of those cells to oxidative stress [15].

Here, we investigated the cellular role of ALADIN by creating two experimental models using the adrenocortical tumour cell line NCI-H295R1, a sub-strain showing characteristics of the glucocorticoid-producing zona fasciculata of the adrenal cortex [16]. Cells were engineered to either over-express or down-regulate *AAAS* by inducible stable transfection. Subsequently, we investigated alterations in steroidogenic gene expression and assessed functional consequences by steroid quantification from cell supernatants employing a liquid chromatography/tandem mass spectrometry (LC/MS-MS) method for simultaneous quantification of 13 key steroids of the adrenal steroidogenic pathway. In addition, we examined the role of ALADIN on cell viability, oxidative stress response and nuclear import of aprataxin, DNA ligase 1 and ferritin heavy chain 1.

## Materials and Methods

### Plasmids and vector construction for short hairpin RNA silencing of gene expression and over-expression

For generation of the *AAAS* shRNA-inducible H295R cell clones, we employed the T-REx system (Invitrogen, Life Technologies, Darmstadt, Germany). Oligonucleotides were designed using the Ambion web-based software “siRNA Target Finder” and compared to the human genome database for no more than 16–17 contiguous base pairs of homology to other coding sequences using NCBI BLAST (www.ncbi.nlm.nih.gov/BLAST). We selected six regions to be the target sequence for shRNA silencing of *AAAS* (V1: NM_015665.5) and used the two best regions (nt279-299 AAATGAAATTGCAAACTCAGA and nt410-430 AAGATCTGATCGCTGAATTTG) for further experiments. As a negative control we used a non-targeting scrambled siRNA (AAAGTACTGCTTACGATACGG). To create pTER plasmids we used two complementary hairpin siRNA template oligonucleotides and ligated into *BglII* and *HindIII* restriction sites of the inducible small-interfering-RNA expression vector pTER, which was generously provided by Enzo Lalli [17]. The inducible pcDNA4/TO expression vector (Invitrogen, Life Technologies) was used for h*AAAS* cDNA over-expression. The full length *hAAAS* cDNA was cloned between *Hind*III and *Xho*I sites into 5’-de-phosphorylated pcDNA4/TO.

Investigation of nuclear import processes was done using C- or N-terminal fluorescent protein vectors (pFP C1 or pFP N3) (Takara Bio Europe/Clonetech, Saint-Germain-en-Laye, France). Full length cDNA of *APTX* (V1: NM_175073.2; V6: NM_001195248.1; V7: NM_001195249.1), *LIG1* (V1: NM_000234.2) and *FTH1* (V1: NM_002032.2) were cloned to create YFP-C1-LIG1, APTX-N3-YFP and FTH1-N3-YFP plasmids. All plasmids constructs were confirmed by DNA sequencing.

### Cell culture and treatments

NCI-H295R1-TR cells containing a tetracycline response system were kindly provided by Enzo Lalli [18,19]. The cells were cultured in DMEM/F12 medium (Lonza, Cologne, Germany) supplemented with 5% Nu-serum (BD Biosciences), 1% insulin-tranferrin-selenium-X supplement (Gibco, Life Technologies), 1% antibiotic-antimycotic (100 U penicillin/ml, 0,1 mg/ml streptomycin sulphate and 0.25 μg/ml amphotericin B, PAA, GE Healthcare Europe GmbH, Freiburg, Germany) and 5 μg/ml blasticidin S (InvivoGen, Toulouse, France). Cells were maintained in a 37°C humidified atmosphere (5% CO_2_).

For generation of NCI-H295R1-TR stably expressing *AAAS* shRNA (NCI-H295R1-TR *AAAS* knock-down), the cells were transfected with the two different pTER*-AAAS*1 and pTER*-AAAS*2 shRNA vectors or the scrambled pTER-shRNA vector as a control cell line (NCI-H295R1-TR scrambled shRNA). For generation of NCI-H295R1-TR stably expressing *hAAAS* cDNA (NCI-H295R1-TR *AAAS* over-expression), the pcDNA4/TO-*AAAS* cDNA was transfected into NCI-H295R1-TR. NCI-H295R1-TR cells stably expressing empty pcDNA4/TO were generated in addition without PCR product insert as a control cell line.

NCI-H295R1-TR cells with *AAAS* knock-down shRNA, scrambled shRNA, *AAAS* over-expression and pcDNA4/TO empty vector were selected and cultured with 100 μg/ml zeocin (InvivoGen, Toulouse, France) in culture media. Doxycyline hydrochloride (tetracycline analogue) (MP Biomedicals, Eschwege, Germany) was used at 1 μg/ml for 48 h to turn on the expression of the subsequent integrated gene or shRNA sequences. The most reliable sub-clones were selected and one clone per construct type was chosen (*AAAS* knock-down shRNA, scrambled shRNA, *AAAS* over-expression and pcDNA4/TO empty vector).

Selected cell lines were cultured in 6-well culture dishes (Corning Costar, Kaiserslautern, Germany) at a density of 2x10^5^ cells/well and treated 24 h after seeding with doxycycline for 48 h. In order to determine efficiency of *AAAS* knock-down, over-expression and expression of genes coding for steroidogenic enzymes total RNA was isolated from one well and used for real-time RT-PCR. In addition, whole protein was isolated from two wells and ALADIN expression was verified on protein level. Steroid production was determined by liquid chromatography tandem mass spectrometry of the cell media of three wells. Experiments were repeated six times.

For analysis of oxidative stress response cells were stimulated with 0.2 mM methyl viologen dichloride (paraquat) (Sigma-Aldrich, Munich, Germany) for 24 h.

### Stable and transient adrenal cell transfection

Cells were cultured in 6-well culture dishes at a density of 1.6x10^5^ cells/well 24 h before subsequent transfections. Cells were transfected using X-tremeGENE HP DNA transfection reagent (Roche Diagnostics, Mannheim, Germany) following the manufacturer’s protocols. All plasmids were used at a concentration of 0.01 μg/μl at an optimised transfection ratio of 2:5 diluted in pure DMEM/F12.

For stable transfections, pTER and pcDNA4/TO constructs were linearised prior transfections by digestion with *PvuI*. At 50% confluency after transfection cells were split 1:4 and sub-cultured in 6-well culture dishes containing medium supplemented with 100 μg/ml zeocin. Clones appeared after approximately 4 weeks and were transferred each into one well of 24-well culture dishes (Corning Costar) by the use of cloning cylinders (Sigma-Aldrich). Clones were grown up and at a confluency of 70% split 1:3.

For transient transfections of pYFP C1-LIG1, APTX-N3 and FTH1-N3, 1.6x10^5^ cells of NCI-H295R1-TR *AAAS* knock-down, scrambled shRNA, *AAAS* over-expression and pcDNA4/TO empty were sub-cultured onto cover slips (Carl Zeiss, Jena, Germany) in 6-well culture dishes. During transfection doxycycline was added to the medium. After 24 h medium was changed and cells were grown for additional 24 h in the presence of doxycycline.

### RNA extraction, cDNA synthesis and quantitative real-time PCR using TaqMan

After seeding, cells were induced with doxycycline for 48 h and total RNA was isolated using the NucleoSpin RNA II kit (Macherey-Nagel, Düren, Germany) according to the protocols from the manufacturer. Purity of the RNA was assessed using Nanodrop Spectrophotometer (ND-1000) (NanoDrop Technologies, Wilmington, DE, USA). RNA was stored at -80°C until use. The amount of 500 ng of total RNA was reverse transcribed using the GoScript Reverse Transcription System (Promega, Mannheim, Germany) following the protocols from the manufacturer and cDNA was stored at -20°C until use. Primers for the amplification of the target sequence were designed using Primer Express 3.0 (Applied Biosystems) and compared to the human genome database for unique binding using BLAST. The primer sequences and gene accession numbers used for the amplification of specific target sequences, which are beta-actin (*ACTB)*, *AAAS*, *CYP11A1*, *CYP17A1*, *CYP21A2*, glutathione reductase (*GSR*), cytochrome P450 reductase (*POR*) and steroidogenic acute regulatory protein (*StAR*), are listed in the Supporting Information of this article (S1 Table).

The qPCR amplifications were performed in triplicates using the GoTaq Probe qPCR Master Mix (Promega) according to the manufacturer’s reaction parameters; using 20 μl total volumes on an ABI 7300 Fast Real-Time PCR System (Applied Biosystems, Life Technologies, Darmstadt, Germany). As housekeeping gene for normalisation *ACTB* was used. Positive controls contained a random mix of cDNA and negative controls contained nuclease-free water instead of cDNA. In all real-time qPCR experiments relative gene expression was calculated using the C_t_ method using standard and semi-log plots of amplification curves. Subsequently, the measured mRNA of each sample was firstly normalised to the gene expression relative of *ACTB* and secondly to a calibrator. In each case the calibrator was the native doxycycline-un-induced sample. Finally, results were expressed either in gene expression relative to *ACTB* or in percentile change in gene expression relative to *ACTB* and calibrator as follows: % change = (C_t_ sample/C_t_ calibrator) *100. In all results repeatability was assessed by standard deviation of triplicate C_t_s and reproducibility was verified by normalising all real-time RT-PCR experiments by the C_t_ of each positive control per run.

The guidelines of the Minimum Information for Publication of Quantitative Real-Time PCR Experiments (MIQE) were followed in this study to allow more reliable interpretation of real-time RT-PCR results [20–22].

### Immunoblots

After induction with doxycycline for 48 h cells were lysed in 100 μl buffer containing 50 mM Tris (pH 7.4), 100 mM NaCl, 4% sodium dodecyl sulfate, and 1% Triton X-100. Protein concentration was measured using D_C_ Protein Assay (BioRad Laboratories, München, Germany) according to manufacturer’s instructions. In order to avoid high viscosity due to high DNA amount an additional step of benzonase nuclease (Novagen Merck Millipore, Darmstadt, Germany) treatment for 30 min at 37°C was done. Before loading 50 μg of protein per lane onto a NUPAGE Bis-Tris Mini Gel (Invitrogen, Life Technologies) samples were prepared with NUPAGE SDS Sample Buffer (4x), NUPAGE Reducing Agent (10x) and deionised water by heating the samples at 70°C for 10 min. After separation and electroblotting onto Amersham Hybond-enhanced chemiluminescence (ECL) nitrocellulose membranes (0.45 μm) (GE Healthcare, Braunschweig, Germany) non-specific binding of proteins to the membrane was blocked by incubation in PBS (Sigma-Aldrich) containing 3% bovine serum albumin (Sigma-Aldrich). The membrane was then probed with primary antibodies either ALADIN Antibody (B-11): sc-374073 (Santa Cruz Biotechnology, Inc., Heidelberg, Germany) (1:100 in 3% BSA) or monoclonal Anti-ß-Actin antibody produced in mouse, clone AC-74 (V1: NP_001092.1) (Sigma-Aldrich) (1:20000 in 3% BSA) over-night at 4°C. Membranes were washed with 0.01% Tween in PBS for 30 min and secondary antibodies goat anti-mouse IgG conjugated to horseradish peroxidase (1:2000 in 3% BSA) or goat anti-rabbit IgG conjugated to horseradish peroxidase (1:3000 in 5% milk powder) were incubated 1 h at room-temperature. Membranes were washed again for 30 min followed by detection using an Amersham ECL Prime Western Blotting detection system (GE Healthcare) using the protocol of the manufacturer. Blots were subsequently exposed to x-ray films and antigen bands visualised by autoradiography using Carestream Kodak autoradiography GPX replenisher/fixer (Kodak, Laborversand A. Hartenstein, Würzburg, Germany).

### Chromatographic and mass spectrometric conditions and sample preparation

Steroids were isolated from 1 ml of cell culture supernatant by liquid/liquid extraction using 3 ml of methyl tertiary butyl ether (MTBE) (Sigma-Aldrich) in borosilicate glass tubes (Fisherbrand, Schwerte, Germany). Samples were vortexed twice for 5 seconds at highest speed, spinned down and subsequently frozen at -20°C for at least 1 h and the top organic layer was poured off into a new clean and dry glass tube. The frozen aqueous layer was discarded. Samples were evaporated for 15 min at 55°C and 0.5 bar using a nitrogen evaporator (Gebrüder Liebisch Labortechnik GmbH, Bielefeld, Germany). Samples were reconstituted in 50/50 methanol/water. They were analysed by tandem mass spectrometry on a Waters Xevo Mass Spectrometer with an acquity uPLC liquid chromatography system as described previously [23]. Steroids were separated using a HSS T3 1.2 x 50 mm column and identified in positive ionisation mode. System set-up is described in Supporting Information of this article (S2 Table). Steroids were quantified by comparison to a calibration series ranging from 0.5 to 1000 ng/ml and set off against total protein content (μg) per well, which represents the cell number. Steroid concentrations were expressed in mMol/g total protein content and each sample was normalised to its native doxycycline-un-induced state giving the percentile change in steroid output as follows: % change = (steroid concentration sample/steroid concentration native doxycycline-un-induced state) *100. All steroids were quantified using a suitable internal standard (S3 Table). A suit of steroids was considered covering the steroid bio-pathway; cortisol, cortisone, 11-deoxycortisol, corticosterone, androstenedione, testosterone, 5α-dihydrotestosterone (DHT), dehydroepiandrosterone (DHEA), 17-hydroxyprogesterone (17OHP), deoxycorticosterone (DOC), progesterone, pregnenolone, and 17-hydroxypregnenolone. Two mass transitions were required to positively quantify each steroid.

### Cell Viability Assay

For measurement of cell viability, stably transfected cells were sub-cultured at a density of 10^4^ cells/well onto 96-well culture dishes (Corning Costar). After 24 h medium was added containing doxycycline. After another 48 h one part of the cells was exposed to oxidative stress with 0.2 mM paraquat. Cell viability was measured in triplicate in paraquat treated and untreated cells using CellTiter-Blue Cell Viability Assay (Promega) according to the manufacturer’s protocol in a final volume of 120 μl. Fluorescence emission at 590 nm of resorufin was measured after 4 h using the Infinite 200 PRO Microplate Reader and the Magellan Data Analysis Software v6.6 (Tecan Group AG, Männedorf, Switzerland). Experiments were repeated at least six times.

### Measurement of total and oxidised glutathione

For measurement of total glutathione (GSH) and its oxidised form (GSSG), stably transfected cells were sub-cultured at a density of 10^4^ cells/well onto white-walled and white-bottomed 96-well culture dishes (Corning Costar). After 24 h medium was added containing doxycycline. After another 48 h cells were exposed to oxidative stress as described above. Total GSH and GSSG were measured in triplicate using GSH/GSSG-Glo Assay (Promega) and the protocol recommended by the manufacturer. Luminescence was recorded after 15 min using the Mithras LB940 luminometer and the MikroWin 2000 software v4.29 (Berthold Technologies, Bad Wildbad, Germany). An integration time of 0.5 sec for each 96-well-micro plate was used. Experiments were repeated at least five times.

### Hydrogen Peroxide Assay

To detect H_2_O_2_ released from and within cells, stably transfected cells were sub-cultured at a density of 10^4^ cells/well onto 96-well culture dishes (Corning Costar). After 24 h medium was added containing doxycycline. After another 48 h the cells were exposed to oxidative stress with 0.2 mM paraquat. The concentration of H_2_O_2_ was measured in triplicate in paraquat treated and untreated cells using Amplex Red Hydrogen Peroxide Assay (Invitrogen) according to the manufacturer’s protocol in a final volume of 100 μl. Amplex Red reagent reacts with H_2_O_2_ and produces the red-fluorescent oxidation product resorufin. Fluorescence emission at 590 nm of resorufin was measured after 5 h using the Infinite 200 PRO Microplate Reader and the Magellan Data Analysis Software v6.6 (Tecan Group AG, Männedorf, Switzerland). Experiments were repeated at least four times.

### Nuclear import processes and microscopy

Transient transfected cells grown on cover slips were washed twice with PBS, fixed with 4% PFA in PBS (Sigma-Aldrich) for 10 min, washed again with PBS and mounted onto 3x1 inch microscope slides (Engelbrecht, Edermünde, Germany) with Vectashield mounting medium for fluorescence without DAPI (Vector Laboratories, Burlingame, CA, USA). For oxidative stress treatment cells were induced for another 24 h with 0.2 mM paraquat before fixation. Fluorescence was recorded using Axiovert 200 M (Carl Zeiss) and pictures taken using x40 objective, Axiocam MRm (Carl Zeiss) and filter set 38HE (excitation BP470/40, beam splitter FT 495, emission BP 525/50). Bright-field and fluorescence pictures were analysed using Fuji v1.48b (Fuji Is Just ImageJ). All input pictures were grey-scaled with auto exposure time for each picture. Cell cytosols and nuclei were analysed semi-automatically. Briefly, each cell cytosol was measured automatically using the auto-threshold feature of ImageJ (Method Triangle) and the subsequent nucleus was marked manually. Region of interests (ROIs) of cytosols and nuclei were extracted on basis of the bright-field picture. These ROIs were transferred to the fluorescence picture and measured, giving the following data values: for fluorescence intensity of cytosol and nucleus each; mean, median, minimum, maximum, area, standard deviation, percentage distribution, ratio (mean_nuc_/mean_cyt_) and for the nuclei, nucleus numbering (in order to verify and double-check the automatic process). Output values for fluorescence intensity of cytosols and nuclei ranged from 0 to 255; consequently, 0 resembles no fluorescence intensity and 255 maximum fluorescence intensity. Ratios mean_nuc_/mean_cyt_ were plotted in order to ascertain potential import impairment. Experiments were repeated twice.

### Statistics

Statistical analyses were made using the open-source-software R i386 v2.15.0 and R Studio version v0.98.1074 (The R Foundation for Statistical Computing, Vienna, Austria). All values were expressed as a mean ± standard deviation. As assumption of normality (“goodness-of-fit”) the following tests were conducted: Shapiro-Wilk-test and Q-Q-Plot. Unpaired and paired Student’s t-tests were used. During evaluation of the results a confidence interval alpha of 95% and P values lower than 0.05 were considered as statistically significant. Results are shown as box plots which give a fast and efficient overview about median, first and third quartile (25^th^ and 75^th^ percentile, respectively), interquartile range (IQR), minimal and maximal values and outliers. Box plots give an intuitive first impression of position and statistical spread of the measurement.

## Results

### Establishment of two different ALADIN expression models

**Fig 1:**
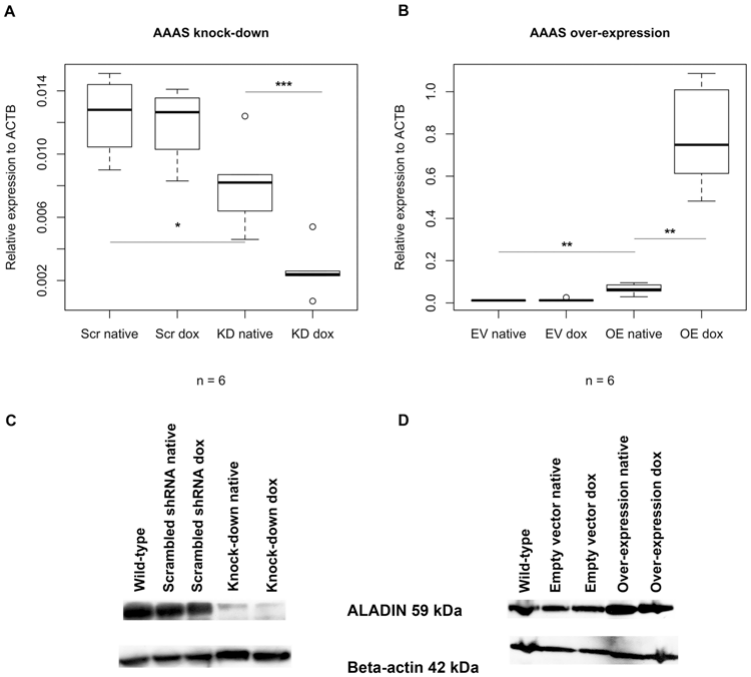
Down-regulation and over-expression of *AAAS* on mRNA and protein level. Knock-down and over-expression in stably transfected NCI-H295R1-TR cells was induced by treatment with doxycycline for 48 h: (A-B) Boxplots of down-regulation and over-expression of *AAAS* on mRNA level (TaqMan analysis) and (C-D) evidence of down-regulation and over-expression of *AAAS* on protein level (Western blot). Scr, scrambled shRNA. KD, knock-down. EV, empty vector (= pcDNA4/TO). OE, over-expression. Native, without doxycycline induction. Dox, doxycycline induction. n, minimum number of independent experiments. P-values: * P<0.05, ** P<0.01, *** P<0.001. Significant differences were measured with paired and unpaired Student’s t-Test. Boxplot widths are proportional to the square root of the samples sizes. Whiskers indicate the range outside 1.5 times the inter-quartile range (IQR) above the upper quartile and below the lower quartile. Outliers were plotted as dots.

Down-regulation and over-expression of *AAAS* was confirmed on mRNA level (n = 6; n gives the minimum number of independent experiments) and on the protein level by Western blot (Fig 1) (n = 6). The establishment of stable doxcycline-inducible ALADIN knock-down and over-expression sub-clones of the adrenocortical cell line NCI-H295R1 was successful.

Without doxycycline stimulation we observed a partial *AAAS* knock-down and over-expression when compared to the subsequent controls (Fig 1). Many of the positive results shown in the article were based on the doxycycline un-induced cells and it had to be assumed that the TREx system used as method of choice appeared to be leaky. The repressor-expressing plasmid pcDNA6TR seemed to be out-numbered in each cell model by the inducible expression vectors pTER for shRNA silencing and pcDNA4/TO for h*AAAS* cDNA over-expression which both contain the tetracycline-response element. In the doxycycline un-induced state the amount of repressor produced seemed not to be enough in order to block expression of shRNA or h*AAAS* cDNA leading to the partial *AAAS* knock-down and over-expression described here.

### Deprivation of ALADIN leads to impairment of glucocorticoid and androgenic steroidogenesis and cytochrome P450 oxidoreductase

**Fig 2:**
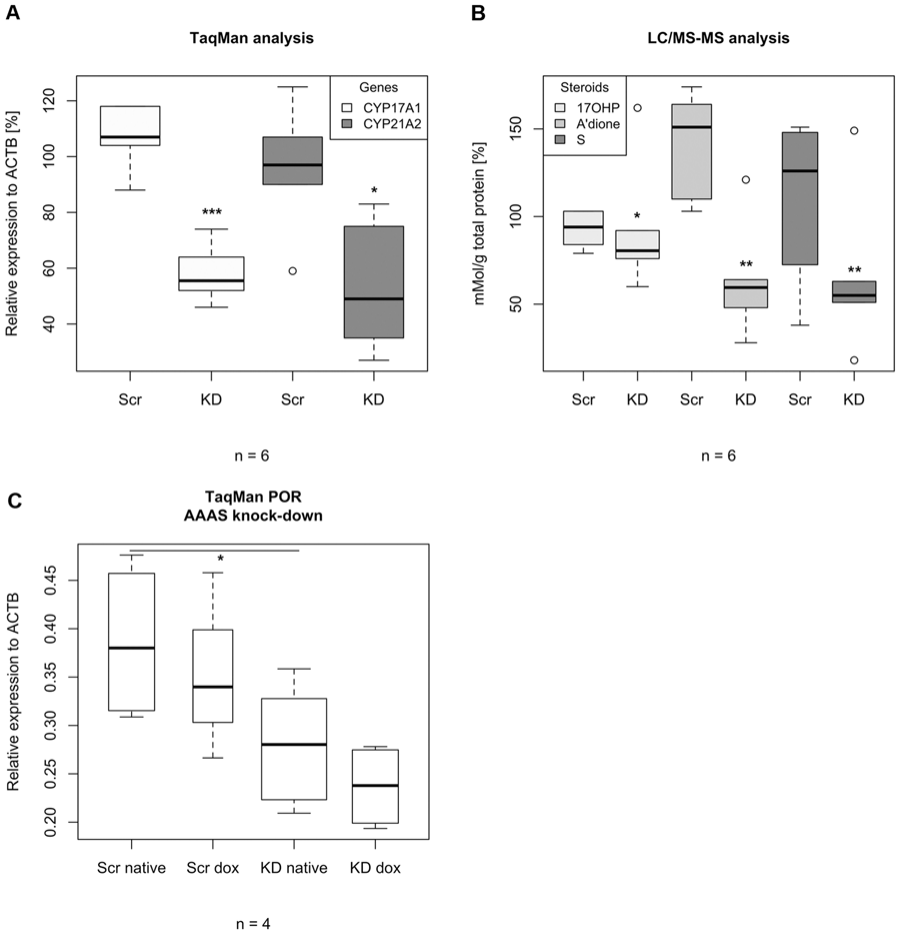
TaqMan analysis and LC/MS-MS of stably transfected NCI-H295R1-TR *AAAS* knock-down cells. Cells were induced by 48 h treatment with doxycycline. Scr, scrambled shRNA. KD, knock-down. 17OHP, 17-hydroxyprogesterone. A’dione, androstenedione. Compound S, 11-deoxycortisol. n, minimum number of independent experiments. P-values: * P<0.05, ** P<0.01, *** P<0.001. Significant differences were measured with paired and unpaired Student’s t-Test. Boxplot widths are proportional to the square root of the samples sizes. Hundred percent boxes for native scrambled shRNA and knock-down are not shown. Whiskers indicate the range outside 1.5 times the inter-quartile range (IQR) above the upper quartile and below the lower quartile. Outliers were plotted as dots.

To investigate the effects of *AAAS* knock-down and over-expression on steroidogenesis we assessed mRNA expression of key enzymes involved in steroidogenesis including *CYP11A1*, *CYP11B1*, *CYP17A1*, *CYP21A2*, 24-dehydrocholesterol reductase (*DHCR24*), cytochrome P450 oxidoreductase (*POR)* and *StAR*. We further measured steroid production employing LC/MS-MS in NCI-H295R1 *AAAS* knock-down and *AAAS* over-expression cells. With quantitative RT-PCR we revealed that *AAAS* knock-down induced a significant down-regulation of the genes coding for enzymes involved in the glucocorticoid and androgenic pathways, i.e. 17α-hydroxylase/17,20lyase (*CYP17A1*) and 21-hydroxylase (*CYP21A2*) (n = 6) (Fig 2). Expression of *StAR*, *CYP11A1*, *CYP11B1* and *DHCR24* was not affected in these cells (S1 and S2 Figs). Similar effects were seen in the functional steroid output of the cells measured by LC/MS-MS. Changes in steroid production were predominantly observed for 17OHP, 11-deoxycortisol (compound S) and androstenedione, whereas only minor changes in the remaining 10 steroids were observed. Thus, for simplification, the following results focus on those three steroids. We revealed that ALADIN knock-down cells show a significant decrease in precursor metabolites required in the glucocorticoid and androgenic pathways. These precursors are 17OHP, 11-deoxycortisol and androstenedione (n = 6) (Fig 2). There was no change in precursor metabolites of the mineralocorticoid pathway (DOC) (S3 Fig). Furthermore we show that the ALADIN-dependent impairment of the glucocorticoid and androgenic pathways is accompanied by a down-regulation of the gene coding for POR, an enzyme managing electron transfer from NADPH to CYP17A1 and CYP21A2 microsomal P450 hydroxylases (Fig 2). A decrease in POR gene expression was observed in knock-down cells prior to doxycycline treatment, most likely representing the partial *AAAS* knock-down shown in Fig 1.

*AAAS* over-expression in at least four triplicate experiments did not result in significant differences in expression of key enzymes involved in steroidogenesis and also did not impact on steroid production (S1, S2 and S3 Figs, respectively).

### The redox homeostasis of the cell is altered by ALADIN knock-down under exogenous oxidative stress

We investigated cell viability in both the *AAAS* knock-down and over-expression models. To determine the role of ALADIN in redox homeostasis, we ascertained how *AAAS* knock-down and over-expression affect the ratio between GSH and GSSG. When cells are exposed to increased levels of oxidative stress, NADPH decreases and GSSG accumulates. Consequently, the GSH/GSSG ratio decreases which is a good indicator for cellular oxidative stress. NADPH is used by glutathione reductase (GSR) to generate the anti-oxidant GSH from its oxidised form GSSG. *GSR* gene expression was measured in both *AAAS* expression models in order to verify a GSR-dependent redox pathway disturbance leading to accumulation of GSSG.

### Cell viability with and without exogenous oxidative stress

**Fig 3:**
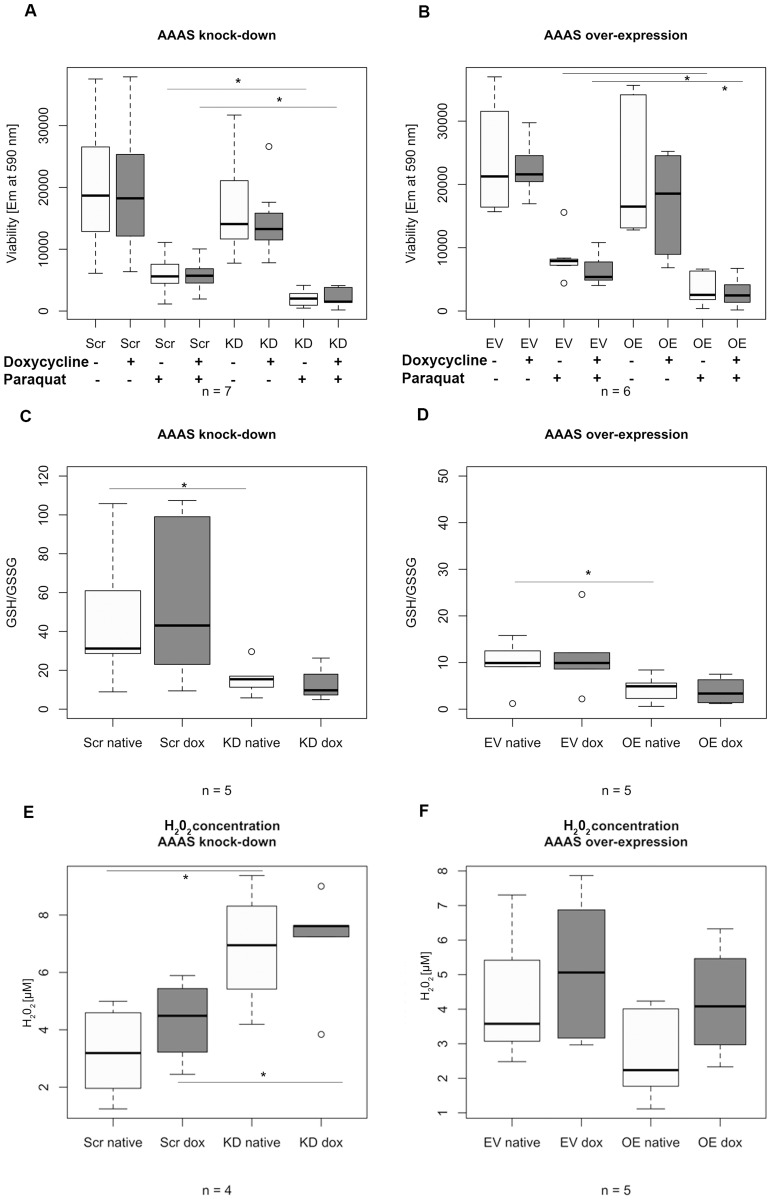
Boxplots of the analysis of (A-B) cell viability, (C-D) glutathione ratios and (E-F) hydrogen peroxide production under oxidative stress. ALADIN knock-down and over-expression in stably transfected NCI-H295R1-TR was induced by 48 h treatment with doxycycline and cells were additionally treated with 0.2 mM paraquat for 24 h. Em, fluorescence emission at 590 nm. GSH, reduced glutathione. GSSG, oxidised disulphide glutathione. H_2_O_2_, hydrogen peroxide. Scr, scrambled shRNA. KD, knock-down. EV, empty vector (= pcDNA4/TO). OE, over-expression. Native, without doxycycline induction. Dox, doxycycline induction. n, minimum number of independent experiments. P-values: * P<0.05. Significant differences were measured with unpaired Student’s t-Test. Boxplot widths are proportional to the square root of the samples sizes. Whiskers indicate the range outside 1.5 times the inter-quartile range (IQR) above the upper quartile and below the lower quartile. Outliers were plotted as dots.

In NCI-H295R1-TR *AAAS* knock-down cells exposed to paraquat for 24 h (n = 7) we observed a significant decrease in cell viability with oxidative stress compared to treated control cells (Fig 3). Viability was not decreased after doxycycline treatment in scrambled shRNA-transfected cells. However, a decrease was observed in knock-down cells prior to doxycycline treatment, most likely representing the partial *AAAS* knock-down shown in Fig 1. The fully induced *AAAS* knock-down did not lead to a further decrease in cell viability after paraquat. Cell viability without oxidative stress was not significantly altered.

Surprisingly, in *AAAS* over-expressing cells we observed similar effects after paraquat treatment (n = 6) (Fig 3). Viability was not altered after doxycycline induction in the control cells carrying the empty vector. However, cell viability was already significantly decreased after paraquat treatment in the native over-expression cells without the induction of doxycycline representing a partial *AAAS* over-expression (Fig 1). The fully induced over-expression cells did not show further alteration in viability after treatment with paraquat. Cell viability without oxidative stress did not show any significant change.

### GSH/GSSG ratio with exogenous oxidative stress

In Fig 3 we demonstrate that GSH/GSSG ratios were markedly decreased in NCI-H295R1-TR *AAAS* knock-down and over-expression cells after paraquat treatment for 24 h compared to treated control cells (n = 5). However, in cells with the partial *AAAS* knock-down and over-expression without induction of doxycycline GSH/GSSG ratios were already significantly down-regulated and no further impairment after doxycycline induction could be observed.

### Hydrogen peroxide production with exogenous oxidative stress

In order to give further evidence that ALADIN is involved in the oxidative stress response of the cell we measured hydrogen peroxide (H_2_O_2_) production in stably transfected cells after exposure to oxidative stress. We presumed that after oxidative stress treatment the oxidants NADPH and GSH will decrease leading to a rise of H_2_O_2_. Cells showing an impaired oxidative response concomitantly suffer from an increased ROS level and cellular damage.

Consistent with the results of cell viability analysis and GSH/GSSG ratio in NCI-H295R1-TR *AAAS* knock-down cells after paraquat treatment we observed a significantly increased level of H_2_O_2_ concentration in these cells compared to paraquat-treated control scrambled shRNA-cells (n = 4) (Fig 3). After doxycycline induction in NCI-H295R1-TR *AAAS* knock-down cells no further significant increase of H_2_O_2_ concentration could be observed; the partial knock-down without induction of doxycycline (Fig 1) is enough to disturb the oxidative response of the cell.

In *AAAS* over-expressing cells with or without doxycycline induction we could not demonstrate significant changes in H_2_O_2_ concentration after paraquat treatment compared to control cells carrying the empty vector (n = 5) (Fig 3).

### Gene expression of glutathione reductase

**Fig 4:**
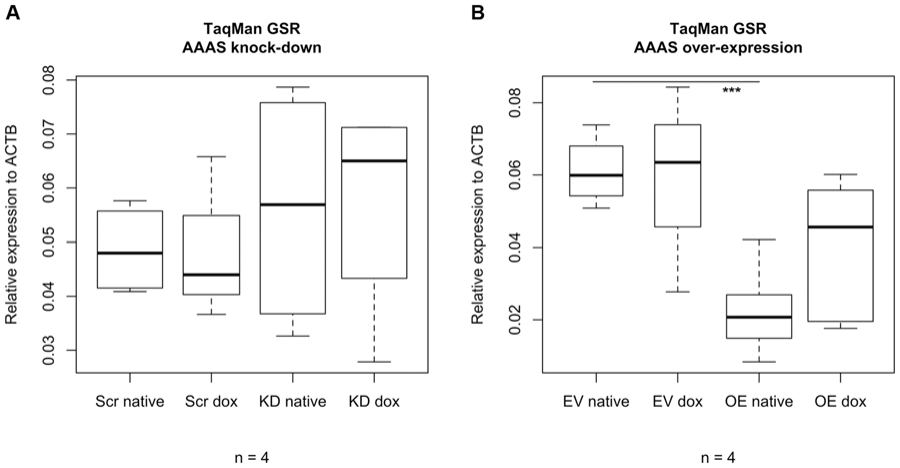
Box plots of TaqMan analysis of glutathione reductase. Stably transfected NCI-H295R1-TR *AAAS* knock-down cells and over-expression were induced by 48 h treatment with doxycycline. GSR, glutathione reductase. Scr, scrambled shRNA. KD, knock-down. EV, empty vector (= pcDNA4/TO). OE, over-expression. Native, without doxycycline induction. Dox, doxycycline induction. n, minimum number of independent experiments. P-values: *** P<0.001. Significant differences were measured with unpaired Student’s t-Test. Boxplot widths are proportional to the square root of the samples sizes. Hundred percent boxes for native scrambled shRNA and knock-down are not shown. Whiskers indicate the range outside 1.5 times the inter-quartile range (IQR) above the upper quartile and below the lower quartile. Outliers were plotted as dots.

Analysis of the gene expression level of glutathione reductase (*GSR*) demonstrated that in NCI-H295R1-TR *AAAS* knock-down cells there was no significant change in expression level compared to scrambled-shRNA control cells with or without doxycycline (n = 4) (Fig 4).

In contrast *GSR* expression level in NCI-H295R1-TR *AAAS* over-expression cells without induction of doxycycline was significantly reduced compared to control cells carrying the empty vector used for the over-expression vector construct (n = 4) (Fig 4). Induction with doxycycline in *AAAS* over-expression cells increases the *GSR* expression level but the increase was not statistically significant.

Overall, we demonstrate that under exogenous oxidative stress using paraquat the redox response of the cell after *AAAS* knock-down in NCI-H295R1-TR is significantly impaired and leads to accumulation of hydrogen peroxide. The expression of *GSR* was not affected in *AAAS* knock-down cells under basal conditions, implying a normal prerequisite of antioxidant defence at *GSR* expression level when applying paraquat. *AAAS o*ver-expression cells suffer from decreased viability and glutathione levels under oxidative stress. In these cells the expression of *GSR* was reduced under basal conditions implying an increase of sensitivity during exogenous oxidative stress.

### The nuclear import of aprataxin, DNA ligase 1 and ferritin heavy chain 1 is disturbed by ALADIN

Using both the human adrenal NCI-H295R1-TR *AAAS* knock-down and over-expression models we investigated the potential impairment of the nuclear import of aprataxin, DNA ligase 1 and ferritin heavy chain 1 employing YFP-specific vectors transiently transfected into the cell lines.

### Nuclear import in *AAAS* knock-down cells

**Fig 5:**
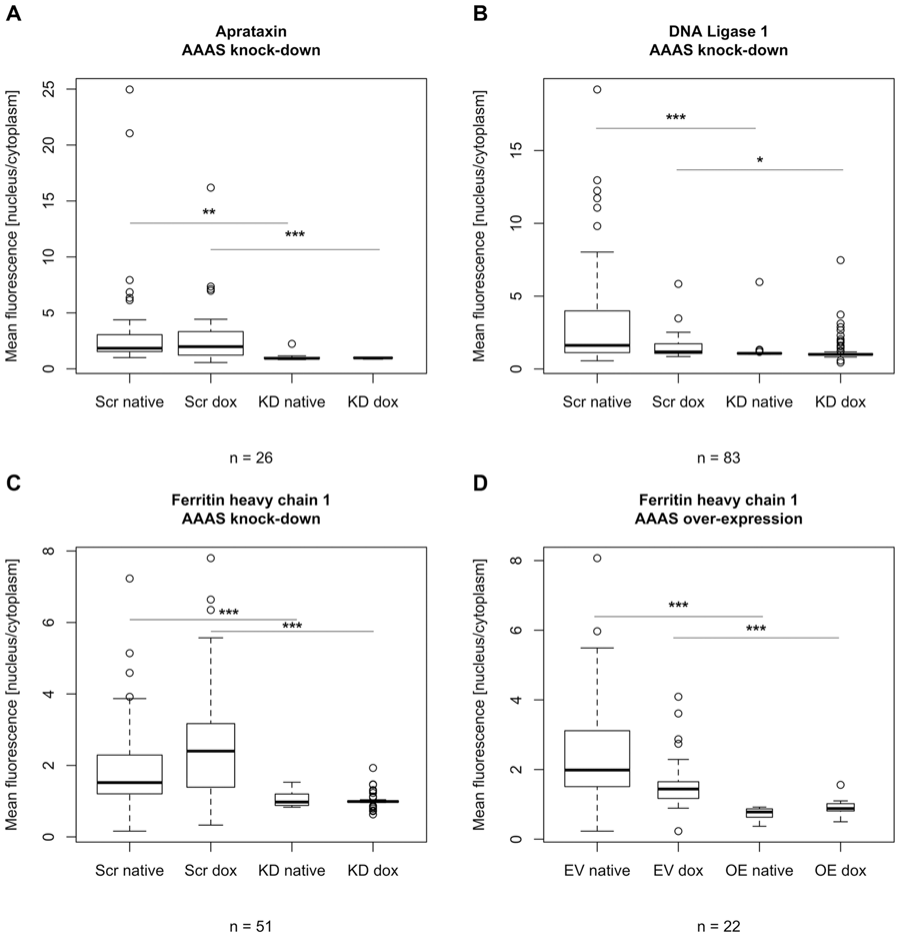
Analysis of fluorescent microscopy of the nuclear import of aprataxin, DNA ligase 1 and ferritin heavy chain 1. Knock-down and over-expression in stably transfected NCI-H295R1-TR *AAAS* knock-down and over-expression cells was induced by 48 h treatment with doxycycline. Scr, scrambled shRNA. KD, knock-down. EV, empty vector (= pcDNA4/TO). OE, over-expression. Native, without doxycycline induction. Dox, doxycycline induction. n, minimum number of analysed cells per cell type. P-values: * P<0.05, ** P<0.01, *** P<0.001. Significant differences were measured with unpaired Student’s t-Test. Boxplot widths are proportional to the square root of the samples sizes. Whiskers indicate the range outside 1.5 times the inter-quartile range (IQR) above the upper quartile and below the lower quartile. Outliers were plotted as dots. The experiment was repeated twice.

**Fig 6:**
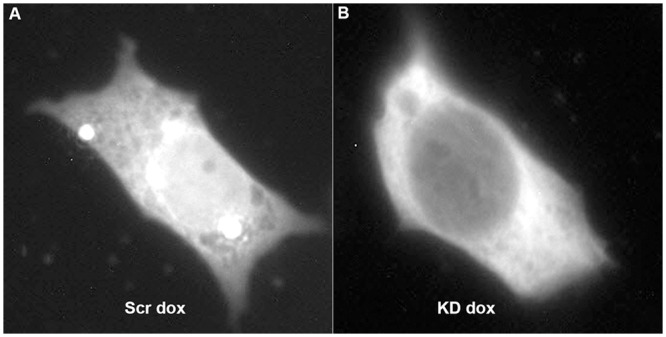
Nuclear import of ferritin heavy chain 1-tagged YFP. Results of fluorescent microscopy of transiently transfected stable NCI-H295R1-TR scrambled shRNA and *AAAS* knock-down cells. Knock-down in stably transfected NCI-H295R1-TR *AAAS* knock-down cells was induced by 48 h treatment with doxycycline. Scr, scrambled shRNA. KD, knock-down. Dox, doxycycline induction.

**Fig 7:**
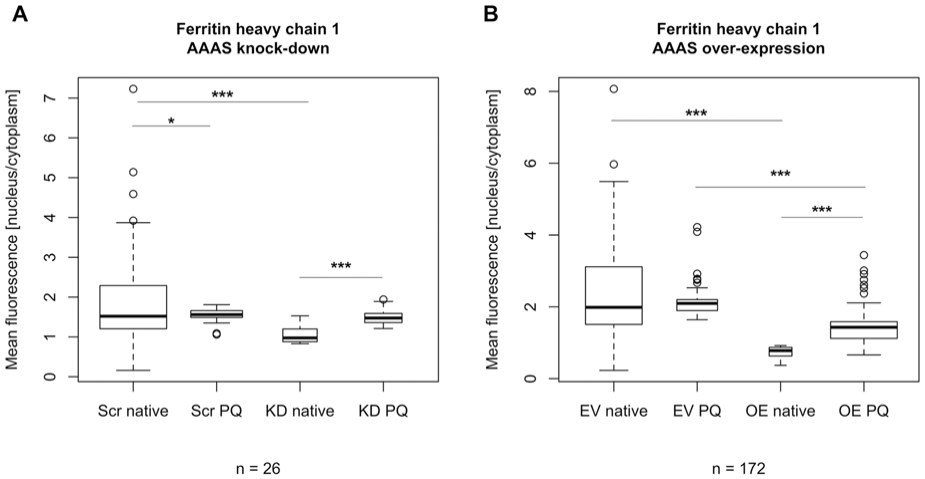
Fluorescent microscopy of the nuclear import of ferritin heavy chain 1 after treatment with paraquat. Knock-down and over-expression in stably transfected NCI-H295R1-TR *AAAS* knock-down and over-expression cells was induced by 48 h treatment with doxycycline. Scr, scrambled shRNA. KD, knock-down. EV, empty vector (= pcDNA4/TO). OE, over-expression. Native, without paraquat treatment. PQ, paraquat treatment. n, minimum number of analysed cells per cell type. P-values: * P<0.05, *** P<0.001. Significant differences were measured with unpaired Student’s t-Test. Boxplot widths are proportional to the square root of the samples sizes. Whiskers indicate the range outside 1.5 times the inter-quartile range (IQR) above the upper quartile and below the lower quartile. Outliers were plotted as dots. The experiment was repeated twice.

The nuclear import of aprataxin, DNA ligase 1 and ferritin heavy chain 1 was markedly impaired after ALADIN deprivation (n = 26, n = 83 and n = 51, respectively; n gives the minimum number of analysed cells per cell type) (Figs 5 and 6). Nevertheless, nuclear import was not changed after doxycycline treatment but already in the native state without doxycycline induction probably reflecting a partial *AAAS* knock-down. The induced *AAAS* knock-down did not lead to a further decrease of nuclear import of aprataxin, DNA ligase 1 and ferritin heavy chain 1 compared to the native state without doxycycline induction (Fig 5). Upon treatment with paraquat for 24 h we found a significant increase in nuclear import of ferritin heavy chain 1 in scrambled-shRNA control cells and *AAAS* knock-down cells but there was no significant difference between those two groups (n = 26) (Fig 7).

### Nuclear import in *AAAS* over-expression cells

Cells over-expressing ALADIN did not show any change in nuclear import of aprataxin and DNA ligase 1 (n = 55 and n = 50, respectively) (S4 Fig). Interestingly, the import of ferritin heavy chain 1 was markedly impaired (n = 22) (Fig 5). The nuclear import of FTH1 was already impaired in the doxycycline-un-induced *AAAS* over-expressing cells and no further impairment could be observed after doxycycline induction. When treating control cells carrying the empty over-expression vector and *AAAS* over-expression cells with 0.2 mM paraquat for 24 h FTH1 was significantly imported into the nucleus (n = 172) (Fig 7). There was a significant difference in FTH import after paraquat treatment between control and over-expressing cells.

## Discussion

We investigated the cellular role of ALADIN by creating two experimental expression systems using the human adrenocortical carcinoma cell line NCI-H295R1-TR that resembles the glucocorticoid producing adrenal zona fasciculata [16]. These cells were successfully transformed to either over-express or down-regulate ALADIN by a doxycycline-inducible stable expression of either *AAAS*-shRNA (*AAAS* down-regulation) or *AAAS*-mRNA (*AAAS* over-expression). In this study we successfully demonstrate that *AAAS* knock-down firstly induces a significant down-regulation of genes coding for type II microsomal CYP enzymes involved in the glucocorticoid and androgen pathways of steroidogenesis, *CYP17A1* and *CYP21A2*, and of the gene coding for their electron donor enzyme POR. Secondly, we revealed that the functional output of these enzymes, i.e. precursor metabolites of the glucocorticoid and androgen pathways, was markedly decreased in ALADIN knock-down cells. In triple A patients adrenal insufficiency may appear later in life than the other two symptoms and its development is gradually [24]. Glucocorticoid and androgen production is affected, consistent with our results *in-vitro* where production of precursors involved in these pathways (17OHP, 11-deoxycortisol and androstenedione) was impaired. 15% of patients however show a deficiency in mineralocorticoid production [25,28]. Precursor metabolites of the mineralocorticoid pathway (DOC) were not impaired in our experiments. Mitochondrial aldosterone synthase encoded by *CYP11B2* exhibits 11ß-hydroxylase activity beside its activity to catalyse aldosterone from corticosterone by 18-hydroxylation. 11ß-hydroxylase activity is expressed by a closely related mitochondrial enzyme CYP11B1 in order to produce cortisol from 11-deoxycortisol [26]. Furthermore it was shown that CYP11B2 exhibits some activity to hydroxylate 11-deoxycortisol [26,27]. Thus, it can be assumed that 11-deoxycortisol can serve as a substrate for aldosterone production. The relative preservation of mineralocorticoid production in triple A patients resembles the lower production of aldosterone compared to cortisol. This may save the zona glomerulosa from cell degeneration as it is seen for the other zones [28].

Even though Prasad et al. already provided some evidence that lentivirally transduced ALADIN-deficient adrenal cells show an alteration in steroidogenesis due to a decrease of expression of steroidogenic acute regulatory protein (StAR) and P450c11ß (CYP11B1) [12], we employed stable transfection for *AAAS* knock-down (and over-expression) and comprehensively investigated the effects on steroidogenic enzyme expression and steroid production. By using tandem mass spectrometry for multiple steroid identification and quantitation in this study we are able to provide strong evidence for a significant functional impairment of the glucocorticoid and androgenic pathways in ALADIN-deficient cells. Our findings has led us a step further to the exact mechanism how ALADIN deficiency affects steroidogenesis and elucidates more details about the pathogenicity in Triple A syndrome. These results therefore significantly add to our understanding of the role of ALADIN for adrenal steroidogenesis. Many of the results are demonstrated in the partial *AAAS* knock-down state, i.e. the doxycycline un-induced state in which too little repressor is produced to efficiently block *AAAS* shRNA expression leading to a premature *AAAS* knock-down. Nevertheless, the amount of *AAAS* shRNA produced is enough to silence *AAAS* gene expression and to exert a pathogenic phenotype in the adrenal cells. Adenoviral transduction in primary bovine adrenocortical cells has been shown to lead to impairment of steroidogenesis and alteration of cell morphology [29]. In regard to human adrenocortical cells Matkovic et al. could show that adenoviral infection leads to alteration in steroidogenesis, presumably not due to viral transgene expression but rather due to viral structure encounter [30]. We therefore assume that the discrepancies in the findings between Prasad et al. and our study lie primarily in the alternative cell model (transduction and stable transfection) and to some extent in the experimental sample sizes especially for steroid analyses. Additionally, the zona fasciculata sub-strain 1 of NCI-H295R cells [16] which resembles one of the zones most degenerated in triple A patients [28] is different from the cells used by Prasad and colleagues.

**Fig 8:**
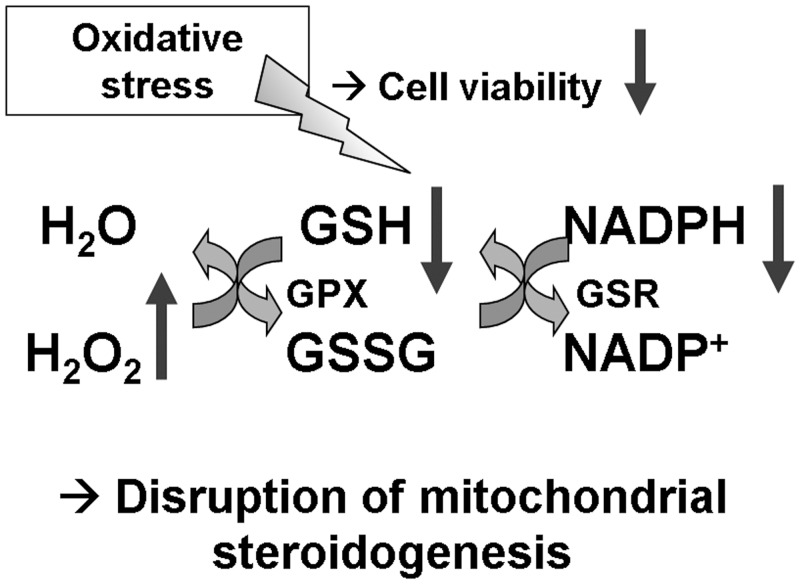
Increase of cellular susceptibility to oxidative stress after *AAAS* knock-down in NCI-H295R1-TR. GSH, reduced glutathione. GSSG, oxidised glutathione disulphide. GPX, glutathione peroxidase. GSR, glutathione reductase. H_2_O_2_, hydrogen peroxide.

In our study we also ascertained that after *AAAS* knock-down in NCI-H295R1-TR cells not only cellular NADPH levels and GSH/GSSG levels under oxidative stress using paraquat are significantly decreased, but also that the concentration of cellular hydrogen peroxide (H_2_O_2_) is increased. Glutathione reductase (*GSR*) expression was not altered under basal conditions suggesting that the increase in sensitivity to oxidative stress in these cells was not the result of low *GSR* expression. These findings imply that ALADIN knock-down cells have an altered redox homeostasis and, hence, are more sensitive to oxidative stress (Fig 8). In order to further address the involvement of ALADIN in the oxidative stress response we studied the impairment of the nuclear import of proteins that protect the nucleus against reactive oxygen species (ROS). The nuclear import of aprataxin, DNA ligase 1 and ferritin heavy chain 1 was markedly decreased after ALADIN depletion. We can therefore conclude that as previously shown in fibroblast cells deriving from triple A syndrome patients [11,14,15] ALADIN is involved in oxidative stress responses and import of these proteins also in adrenal tissue. In addition, we previously observed that fibroblast cells of triple A patients show an altered down-regulation or induction of genes associated with oxidative stress and antioxidant defence [13]. Aprataxin and DNA ligases work concomitantly together in order to initiate and repair DNA in multiple pathways and upon multiple damages, especially ROS-mediated damage [31]. Furthermore it has previously been shown that neurological disorders caused by mutations in the gene *APTX* coding for aprataxin are probably caused by accumulation of unrepaired DNA strand breaks resulting from abortive DNA ligation, generated especially by oxidation [31,32]. Another possibility of abortive DNA ligation (including DNA ligase 1) is during ribonucleotide excision repair. It has been shown that adenylated RNA-DNA lesions are resolved by aprataxin and that accumulation of these lesions is associated with neurologic diseases [33]. Triple A syndrome is commonly associated with a variety of neurologic impairments, presumably due to an increasing number of RNA-DNA lesions in patients’ neurons. Aprataxin and DNA ligase 1 both contain a nuclear localisation signal and are thought to be translocated into the nucleus by a karyopherin-α/β-mediated pathway through NPCs. Hirano et al. showed that nuclear import of aprataxin and DNA ligase 1 is impaired when treating patient fibroblasts and control cells with L-buthionine-(S,R)-sulfoximine (BSO), a glutathione-depleting agent, but single-stranded DNA breaks increased only in patient cells [15]. Increased oxidative stress by BSO seems to intensify nuclear import impairment in patient fibroblasts seen by accumulation of single-stranded DNA breaks. These findings in patient fibroblasts are now confirmed by our results in ALADIN knock-down adrenal cells which are also depleted of reduced glutathione and show impaired nuclear import of aprataxin and DNA ligase 1.

In *AAAS* over-expressing cells, neither steroidogenic enzyme expression nor steroid production was altered. Surprisingly, similar to ALADIN deprivation, over-expressing cells showed a significant impairment of cell viability and GSH/GSSG ratio under oxidative stress but cellular H_2_O_2_ concentration was not increased. In addition we found that over-expressing cells have a decreased expression of *GSR* which explains the decreased GSH/GSSG ratio. These results suggest that *AAAS* over-expression cells do not have an impaired redox potential and are able to detoxify an increased level of ROS after paraquat treatment compared to *AAAS* knock-down cells. In addition, it explains the finding that *AAAS* over-expression cells do not show an alteration in steroidogenesis but rather behave like control cells. The impaired cell viability and decreased expression of *GSR* may be caused by cell stress due to an increased ALADIN protein synthesis and significant accumulation in the cytosol and ER. Cellular ER stress has been recently reported to cause increased protein folding-stress. Initial protein maturation takes place at the ER and is of high importance of proper protein folding. Increased protein synthesis and folding, especially in specialised secretory cells, introduces a high level of stress and a particular challenge for the cell. If the ER machinery is working over-strained an excess of synthesised proteins will be sensed causing a stop of translation, ultimately leading to cell death [34]. We hypothesise that this phenomenon accounts for our *AAAS* over-expressing cells.

*AAAS* over-expressing cells did not show any change in nuclear import of aprataxin or DNA ligase 1. In addition, the import of ferritin heavy chain 1 was significantly decreased after *AAAS* over-expression. These results together with the ALADIN knock-down results confirm our previously reported finding that ALADIN interacts with and is involved in the nuclear import of ferritin heavy chain 1 [14]. Ferritin heavy chain 1 is an iron chelator and its intrinsic ferroxidase activity is an important player in the cellular antioxidant defence system by converting ferrous iron to its ferric storage form which can act in the Fenton reaction as a ROS scavenger. In addition, FTH1 is shown to have transcriptional regulatory activity which explains its translocation to the nucleus besides protecting the DNA from ROS. Regarding its nuclear import it is known that FTH1 enters the nucleus through NPCs but no obvious nuclear localisation signal has been found so far. It is rather thought to be shuttled by a chaperon molecule to the NPC and into the nucleus [35–37]. In corneal epithelial cells it was shown that FTH1 not passively diffuses into the nucleus but is rather shuttling by a chaperon molecule, called ferritoid. But this molecule seems to be highly specific for this cell type [38,39]. In this study that we demonstrate that *AAAS* over-expression leads to a significant accumulation of ALADIN in the cytoplasm; most likely due to a lack of binding sites of ALADIN at the pore complexes. Consequently, mis-localisation of ALADIN in the cytoplasm leads to a decrease of nuclear import and subsequent retention of ferritin heavy chain 1 in the cytoplasm. After treating ALADIN knock-down and over-expression cells with paraquat for 24 h we observed an increase of nuclear import of FTH1. However, this import was significant diminished in the over-expression model in comparison to paraquat treated control cells. In the knock-down model this effect was not obvious, probably due to an insufficient total loss of ALADIN at the nuclear pore. Thus, we show here that ferritin heavy chain 1 is withheld in the cytoplasm by ALADIN in the over-expression cells. It still remains to be clarified how FTH1 is translocated into the nucleus and how this protein interacts with ALADIN.

In conclusion, the results of the knock-down experiments in the human cell line NCI-H295R1 are in accordance with the pathology in triple A syndrome patients showing an alteration in adrenal steroidogenesis, especially the glucocorticoid and androgenic pathways, and an impairment in the cellular response to oxidative stress. No such adrenal phenotype has so far been shown in *Aaas* ^-/-^ mice which predominantly have a zona glomerulosa [40]. Our findings suggest that alteration in adrenal steroidogenesis in humans through POR is caused by a defective redox potential and homeostasis due to ALADIN deficiency. The presence of ALADIN seems to be of high significance for detoxification of ROS in adrenocortical cells and puts weight on the genuine susceptibility of this organ to oxidative stress. Our results demonstrate that these processes especially affect adrenal cells and are likely to be causally linked to the adrenal phenotype in triple A syndrome. Further studies are needed to clarify how the ALADIN-mediated changes in steroidogenesis are coupled to the mitochondrial oxidative stress response and the nuclear import of proteins protecting cells against increased ROS.

## Supporting Information

S1 Fig Boxplots of the results of TaqMan analysis. (A-B) *StAR* and *CYP11A1* of *AAAS* knock-down and over-expression cells, (C) *CYP17A1* and *CYP21A2* and (D) *POR* of *AAAS* over-expression cells. Knock-down and over-expression in stably transfected NCI-H295R1-TR cells was induced by 48 h treatment with doxycycline. Scr, scrambled shRNA. KD, knock-down. EV, empty vector (= pcDNA4/TO). OE, over-expression. Native, without doxycycline induction. Dox, doxycyline induction. n, number of independent experiments. Boxplot widths are proportional to the square root of the samples sizes. Whiskers indicate the range outside 1.5 times the inter-quartile range (IQR) above the upper quartile and below the lower quartile. Outliers were plotted as dots.
(TIF)

S2 Fig Boxplots of the results of quantitative real-time PCR. (A-B) *DHCR24* and (C-D) *CYP11B1* of *AAAS* knock-down and over-expression cells. Knock-down and over-expression in stably transfected NCI-H295R1-TR cells was induced by 48 h treatment with doxycycline. Scr, scrambled shRNA. KD, knock-down. EV, empty vector (= pcDNA4/TO). OE, over-expression. Native, without doxycycline induction. Dox, doxycyline induction. n, number of independent experiments. Boxplot widths are proportional to the square root of the samples sizes. Whiskers indicate the range outside 1.5 times the inter-quartile range (IQR) above the upper quartile and below the lower quartile. Outliers were plotted as dots.
(TIF)

S3 Fig Boxplots of the results of LC/MS-MS. (A) DOC of stably transfected NCI-H295R1-TR *AAAS* knock-down and over-expression cells and (B) 17OHP, a’dione and compound S of *AAAS* over-expression cells. Induction with doxycycline was done for 48 h. Scr, scrambled shRNA. KD, knock-down. EV, empty vector (= pcDNA4/TO). OE, over-expression. DOC, deoxycorticosterone. 17OHP, 17-hydroxyprogesterone. A’dione, androstenedione. Compound S, 11-deoxycortisol. n, number of independent experiments. Boxplot widths are proportional to the square root of the samples sizes. Whiskers indicate the range outside 1.5 times the inter-quartile range (IQR) above the upper quartile and below the lower quartile. Outliers were plotted as dots.
(TIF)

S4 Fig Analysis of fluorescent microscopy of stably transfected NCI-H295R1-TR AAAS over-expression cells. Nuclear import of (A) aprataxin and (B) DNA ligase 1. Induction with doxycycline was done for 48 h treatment. EV, empty vector (= pcDNA4/TO). OE, over-expression. Native, without doxycycline induction. Dox, doxycycline induction. n, minimum number of analysed cells per cell type. Boxplot widths are proportional to the square root of the samples sizes. Whiskers indicate the range outside 1.5 times the inter-quartile range (IQR) above the upper quartile and below the lower quartile. Outliers were plotted as dots. The experiment was repeated twice.
(TIF)

S1 Protocol Quantitative real-time PCR using a double-stranded DNA-binding dye as reporter. (DOC)

S1 Table Real-time qPCR primer sequences. (DOC)

S2 Table LC/MS system parameters. (DOC)

S3 Table Mass transitions and retention times of steroids. Steroids in italic represent internal standards.
(DOC)
