# Supplementary figures and images for: Role of ALADIN in Human Adrenocortical Cells for Oxidative Stress Response and Steroidogenesis
Source: PLoS One. 2015 Apr 13;10(4):e0124582. doi: 10.1371/journal.pone.0124582 (PMC4395102; doi:10.1371/journal.pone.0124582)

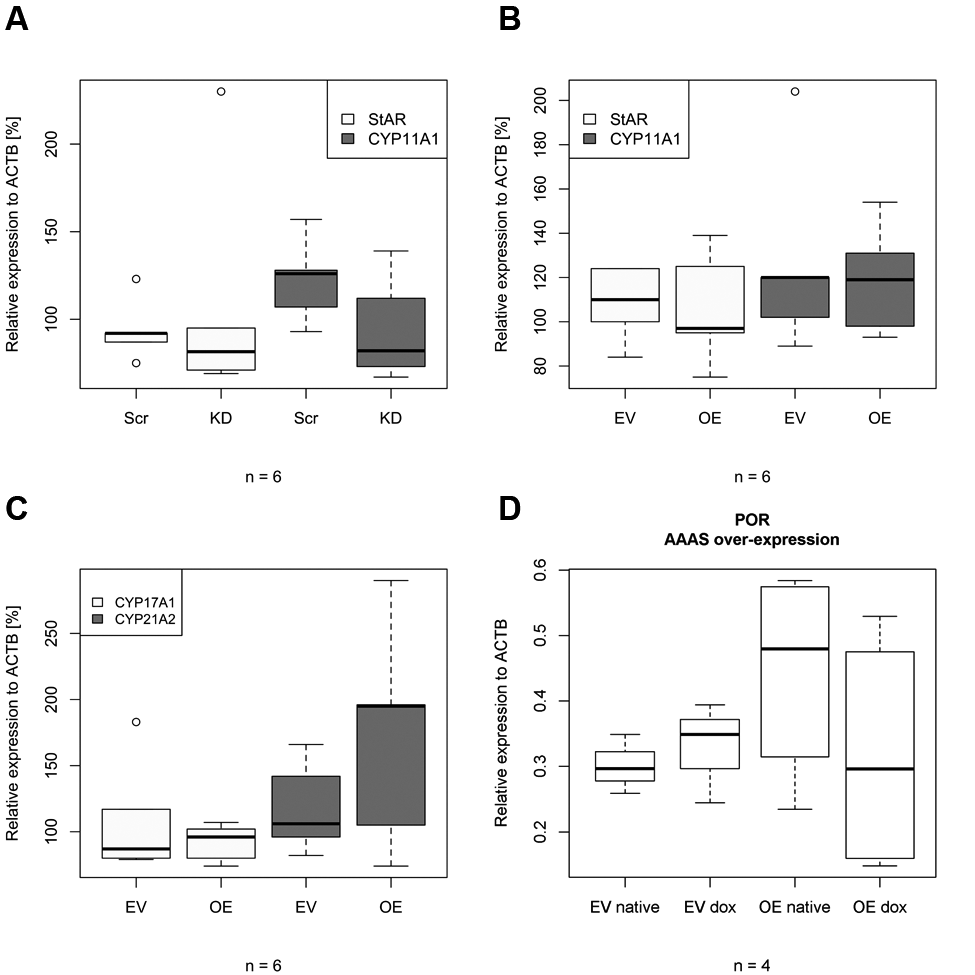

Supplement: S1 Fig — (A-B) StAR and CYP11A1 of AAAS knock-down and over-expression cells, (C) CYP17A1 and CYP21A2 and (D) POR of AAAS over-expression cells. Knock-down and over-expression in stably transfected NCI-H295R1-TR cells was induced by 48 h treatment with doxycycline. Scr, scrambled shRNA. KD, knock-down. EV, empty vector (= pcDNA4/TO). OE, over-expression. Native, without doxycycline induction. Dox, doxycyline induction. n, number of independent experiments. Boxplot widths are proportional to the square root of the samples sizes. Whiskers indicate the range outside 1.5 times the inter-quartile range (IQR) above the upper quartile and below the lower quartile. Outliers were plotted as dots. (TIF) [file pone.0124582.s001.tif]

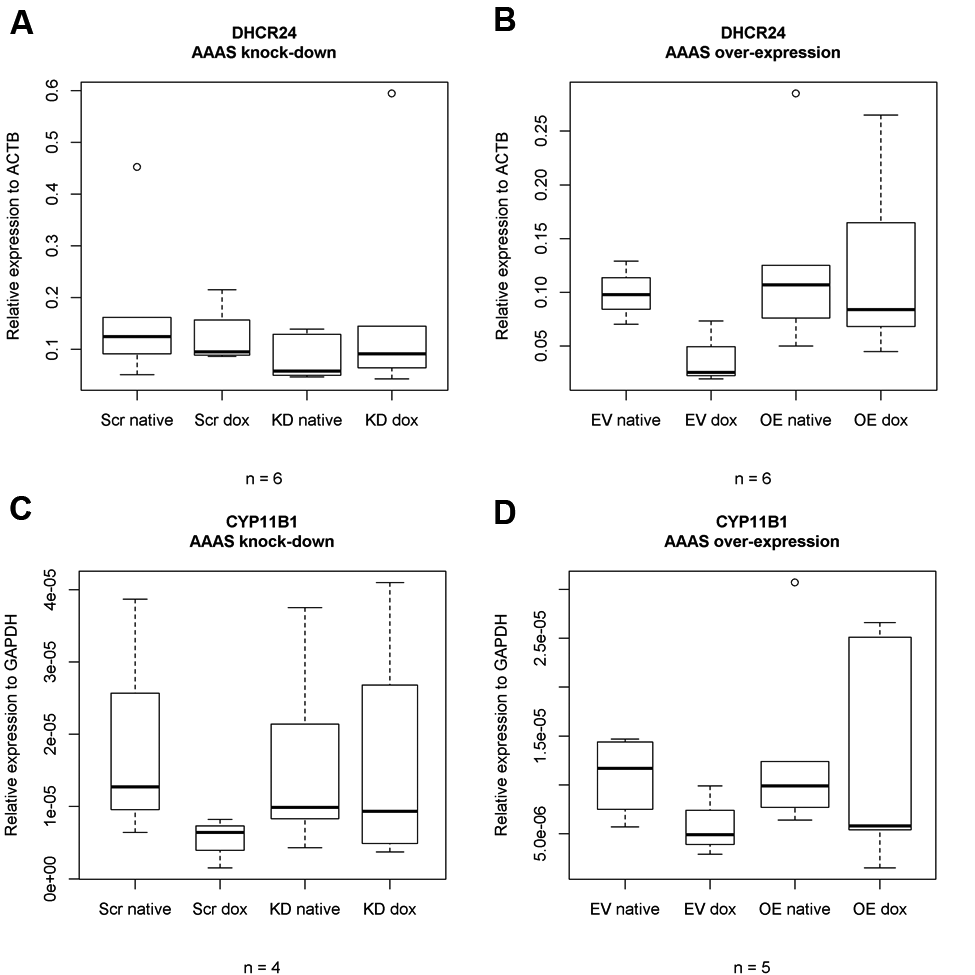

Supplement: S2 Fig — (A-B) DHCR24 and (C-D) CYP11B1 of AAAS knock-down and over-expression cells. Knock-down and over-expression in stably transfected NCI-H295R1-TR cells was induced by 48 h treatment with doxycycline. Scr, scrambled shRNA. KD, knock-down. EV, empty vector (= pcDNA4/TO). OE, over-expression. Native, without doxycycline induction. Dox, doxycyline induction. n, number of independent experiments. Boxplot widths are proportional to the square root of the samples sizes. Whiskers indicate the range outside 1.5 times the inter-quartile range (IQR) above the upper quartile and below the lower quartile. Outliers were plotted as dots. (TIF) [file pone.0124582.s002.tif]

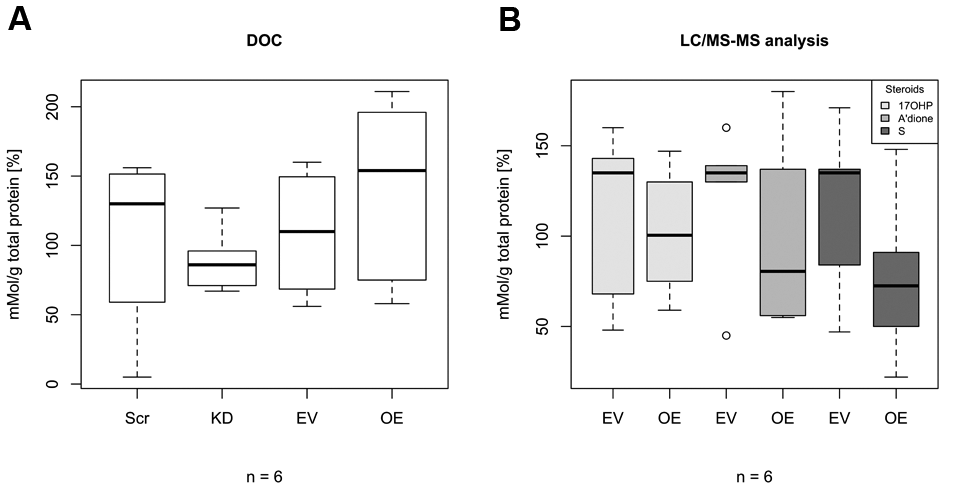

Supplement: S3 Fig — (A) DOC of stably transfected NCI-H295R1-TR AAAS knock-down and over-expression cells and (B) 17OHP, a’dione and compound S of AAAS over-expression cells. Induction with doxycycline was done for 48 h. Scr, scrambled shRNA. KD, knock-down. EV, empty vector (= pcDNA4/TO). OE, over-expression. DOC, deoxycorticosterone. 17OHP, 17-hydroxyprogesterone. A’dione, androstenedione. Compound S, 11-deoxycortisol. n, number of independent experiments. Boxplot widths are proportional to the square root of the samples sizes. Whiskers indicate the range outside 1.5 times the inter-quartile range (IQR) above the upper quartile and below the lower quartile. Outliers were plotted as dots. (TIF) [file pone.0124582.s003.tif]

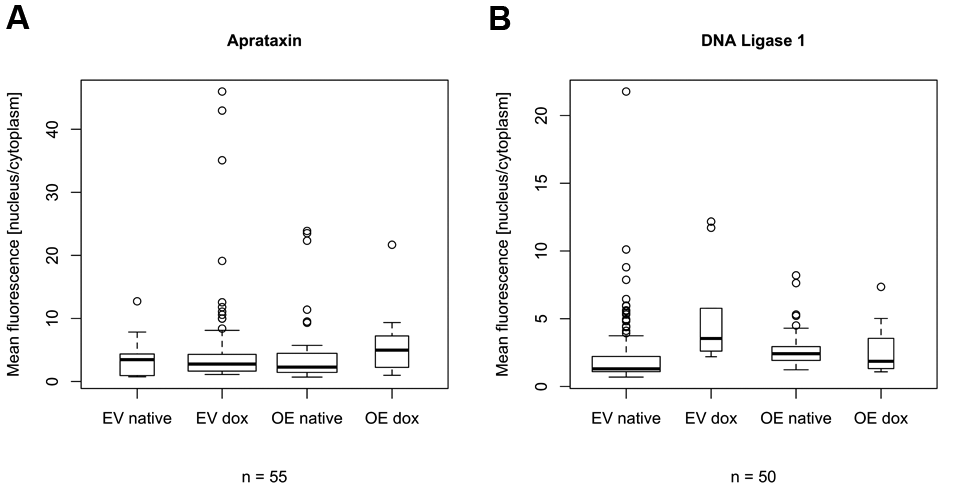

Supplement: S4 Fig — Nuclear import of (A) aprataxin and (B) DNA ligase 1. Induction with doxycycline was done for 48 h treatment. EV, empty vector (= pcDNA4/TO). OE, over-expression. Native, without doxycycline induction. Dox, doxycycline induction. n, minimum number of analysed cells per cell type. Boxplot widths are proportional to the square root of the samples sizes. Whiskers indicate the range outside 1.5 times the inter-quartile range (IQR) above the upper quartile and below the lower quartile. Outliers were plotted as dots. The experiment was repeated twice. (TIF) [file pone.0124582.s004.tif]
