# Supplementary material for: Role of ALADIN in Human Adrenocortical Cells for Oxidative Stress Response and Steroidogenesis
Source: PLoS One. 2015 Apr 13;10(4):e0124582. doi: 10.1371/journal.pone.0124582 (PMC4395102; doi:10.1371/journal.pone.0124582)
Supplement: S1 Protocol — (DOC) [file pone.0124582.s005.doc]

**S1 Protocol. Quantitative real-time PCR using a double-stranded DNA-binding dye as reporter.**

The primer sequences used for the amplification of specific target sequences, which are *CYP11B1*, 24-dehydrocholesterol reductase (*DHCR24*) and glyceraldehyde 3-phosphate dehydrogenase (*GAPDH*) are listed in the Supporting Information of this article (S1 Table).

The qPCR amplifications were performed in duplicate using the GoTaq qPCR Master Mix (Promega) according to the manufacturer’s reaction parameters; using 20 µl total volumes on an ABI 7300 Fast Real-Time PCR System (Applied Biosystems, Life Technologies, Darmstadt, Germany). As housekeeping gene for normalisation *GAPDH* was used. In all real-time qPCR experiments relative gene expression was calculated using the C_t_ method. Results were expressed in gene expression relative to *GAPDH*. In all results repeatability was assessed by standard deviation of duplicate C_t_s and reproducibility was verified by normalising all real-time qPCR experiments by the C_t_ of each positive control per run.
