# Supplementary material for: Role of ALADIN in Human Adrenocortical Cells for Oxidative Stress Response and Steroidogenesis
Source: PLoS One. 2015 Apr 13;10(4):e0124582. doi: 10.1371/journal.pone.0124582 (PMC4395102; doi:10.1371/journal.pone.0124582)
Supplement: S1 Table — (DOC) [file pone.0124582.s006.doc]

**S1 Table. Real-time qPCR primer sequences.**

| Gene | Amplification length | Primer name | Primer sequence |
| --- | --- | --- | --- |
| *ACTB*  (V1: NM_001101.3) | 189 bp | Hs-ß-Actin-F  hs-ß-Actin-R  hs-ß-Actin-Sonde | GCACCCAGCACAATGAAGATC  CGCAACTAAGTCATAGTCCGC  TGCTCCTCCTGAGCGCAAGTACTCC |
| *AAAS*  (V1: NM_015665.5) | 71 bp | 281AAAS-Ex7-F  282AAAS-Ex8-R  283AAAS-hSonde | CCCTACCTCCTTGTCTACCCG  CAGGTGTATGCCCAGGGTG  TCTTCTGGCTGTGCCCAAGTGCTGTC |
| *CYP11A1*  (V1: NM_0007812.2; V2: NM_0001099773.1) | 92 bp | hCYP11A1-F  hCYP11A1-R  hCYP11A1-Sonde | CGGGCTCCGGAAATTACTC  CTGGCGCTCCCCAAAAAT  TTCCGCTTTGCCTTTGAGTCCATCACT |
| *CYP11B1*  (V1: NM_000497.3; V2: NM_001026213.1) | 81 bp | h-CYP11B1-F  h-CYP11B1-R | TGAGGACCTGCACCTGGAAG  ATGCCTGCTCCTCCCAARTC |
| *CYP17A1*  (V1: NM_000102.3) | 97 bp | hCYP17A1-F  hCYP17A1-R  hCYP17A1-Sonde | TGCTTATTAAGAAGGGCAAGGACTT  GAGTCAGCGAAGGCGATACC  TAGAGTTGCCATTTGAGGCCGCCC |
| *CYP21A2*  (V1: NM_000500.7; V2: NM_001128590.3) | 74 bp | hCYP21A2-Ex6F  hCYP21A2-Ex7R  hCYP21A2-Ex6/7-Sonde | CATAGAGAAGAGGGATCACATCGT  TCCACTGGCCTGCCACG  CTCTCCTTGTGCTGCCTCAGCTGCAT |
| *DHCR24*  (V1: NM_014762.3) | 69 bp | DHCR24-F  DHCR24-R  DHCR24-Sonde | CTGGCTGATGGCAGCTTTGT  CCAGGGTACGGCATAGAACAG  CGATGCACTCCGTCCGAAAACTCAGA |
| *GAPDH*  (V1: NM_002046.4; V2: NM_001256799.1) | 75 bp | GAPDH-F  GAPDH-R | GCACCGTCAAGGCTGAGAAC  AGGGATCTCGCTCCTGGAA |
| *GSR*  (V1: NM_000637.3; V2: NM_001195102.1; V3: NM_001195103.1; V4: NM_001195104.1) | 71 bp | GSR-F  GSR-R  GSR-Sonde | TATGCCCTCCACCCCTCAT  TGAAAAAATCCATCGCTGGTT  CAGATCCCCGGTGCCAGCTTAGGA |
| *POR*  (V1: NM_000941.2) | 92 bp | HPOR-F  hPOR-R  hPOR-Sonde | TTTGTGGAAAAGATGAAGAAAACG  GACAGGCGGTTGGCAAACT  AACATCATCGTGTTCTACGGCTCCCAGA |
| *STAR*  (V1: NM_000349.2) | 98 bp | HSTAR-F  hSTAR-R  hSTAR-Sonde | CAGACTTCGGGAACATGCCT  CTTAGAGGGACTTCCAGCCAA  ACCGTGCTCCGCCCTGATGACA |
