# Supplementary material for: Role of ALADIN in Human Adrenocortical Cells for Oxidative Stress Response and Steroidogenesis
Source: PLoS One. 2015 Apr 13;10(4):e0124582. doi: 10.1371/journal.pone.0124582 (PMC4395102; doi:10.1371/journal.pone.0124582)
Supplement: S2 Table — (DOC) [file pone.0124582.s007.doc]

**S2 Table. LC/MS system parameters.**

| LC/MS system set-up | Criteria |
| --- | --- |
| System | Waters Xevo mass spectrometer |
| uPLC | Acquity uPLC system |
| Column | HSS T3, 1.8 µm, 1.2x50 mm column |
| Column Temperature | 60 °C |
| Source | Electrospray source |
| Ionisation Mode | Positive |
| Capillary Voltage | 0.8-1.5 kV (depending on most recent tune file) |
| Cone Voltage | 14-30 V |
| Collision Energy | 8-26 eV (depending on the mass transition) |
| Source Temperature | 150 °C |
| Desolvation Temperature | 600 °C |
| Injection Volume | 20 µL |
| Solvent A1 | Water (LC/MS grade) 0.1 % formic acid |
| Solvent B1 | Methanol (LC/MS grade) 0.1 % formic acid |
| Strong wash | Solvent B |
| Weak wash | 50/50 methanol/water |
| Seal wash | 10 % methanol |
| Solvents A2 and B2 | 50/50 methanol/water |
| Flow rate | 0.6 ml/min |
