# Supplementary material for: Role of ALADIN in Human Adrenocortical Cells for Oxidative Stress Response and Steroidogenesis
Source: PLoS One. 2015 Apr 13;10(4):e0124582. doi: 10.1371/journal.pone.0124582 (PMC4395102; doi:10.1371/journal.pone.0124582)
Supplement: S3 Table — Steroids in italic represent internal standards. (DOC) [file pone.0124582.s008.doc]

**S3 Table. Mass transitions and retention times of steroids. Steroids in italic represent internal** **standards.**

| Steroid | Abbreviation | Quantifier transition  precursor > product | Qualifier transition  precursor > product |
| --- | --- | --- | --- |
| *Cortisol-d4* | *F-d4* | *367 > 121* | *367 > 331* |
| Cortisol | F | 363 > 121 | 363 > 97 |
| Cortisone | E | 361 > 163 | 361 > 121 |
| *Corticosterone-d8* | *B-d8* | *355 > 125* | *355 > 100* |
| Corticosterone | B | 347 > 121 | 347 > 97 |
| *11-deoxycortisol-d2* | *S-d2* | *349 > 97* | *349 > 109* |
| 11-deoxycortisol | S | 347 > 97 | 347 > 109 |
| *Testosterone-d3* | *Test-d3* | *292 > 109* | *292 > 97* |
| Androstenedione | A’dione | 287 > 97 | 287 > 109 |
| Testosterone | Test | 289 > 97 | 289 > 109 |
| *Dihydrotestosterone-d3* | *DHT-d3* | *294 > 258* | *294 > 159* |
| Dihydrotestosterone | DHT | 291 > 255 | 291 > 159 |
| *Dehydroepiandrosterone-d6* | *DHEA-d6* | *277 > 219* | *277 > 259* |
| Dehydroepiandrosterone | DHEA | 289 > 253 | 289 > 271 |
| *17-hydroxyprogesterone-d8* | *17OHP-d8* | *339 > 100* | *339 > 113* |
| Deoxycorticosterone | DOC | 331 > 109 | 331 > 97 |
| 17-hydroxyprogesterone | 17OHP | 331 > 97 | 331 > 109 |
| *Progesterone-d9* | *Prog-d9* | *324 > 100* | *342 > 113* |
| Progesterone | Prog | 315 > 97 | 315 > 109 |
| *Pregnenolone-d4* | *Preg-d4* | *321 > 303* | *321 > 285* |
| Pregnenolone | Preg | 317 > 299 | 317 > 281 |
| *17-hydroxypregnenolone-d3* | *17OHPreg-d3* | *336 > 300* | *336 > 105* |
| 17-hydroxypregnenolone | 17OHPreg | 297 > 279 | 297 > 159 |
